# *“AHead Start or a Pain in theNeck?”*—Establishment and Evaluation of a Video-Based “Hands-On” Head and Neck Ultrasound Course

**DOI:** 10.3390/diagnostics12051239

**Published:** 2022-05-16

**Authors:** Lukas Pillong, Alessandro Bozzato, Dietmar Hecker, Victoria Bozzato, Bernhard Schick, Philipp Kulas

**Affiliations:** Department for Otorhinolaryngology and Head and Neck Surgery, Saarland University Medical Center, Kirrbergerstraße 100, 66421 Homburg, Germany; lukas.pillong@uks.eu (L.P.); alessandro.bozzato@uks.eu (A.B.); dietmar.hecker@uks.eu (D.H.); victoria.bozzato@uks.eu (V.B.); bernhard.schick@uks.eu (B.S.)

**Keywords:** head and neck imaging, head and neck ultrasound, video-based teaching

## Abstract

The COVID-19 pandemic has strongly highlighted the need for more digitalization in healthcare. Teaching ultrasound skills in online courses is a key challenge in this context. The aim of this study was to establish an online video-based head and neck ultrasound course with an evaluation of the quality, effectiveness, and feasibility of this teaching method compared to in-person teaching. Twenty-two medical students were taught head and neck ultrasound in two groups: one group in an in-person course and the other one in a video-based course. Learning success was analyzed using self-evaluation forms and external assessment by an experienced ultrasonographer. Comparing pre- and post-training self-evaluation, all participants showed statistically significant learning progress. In the external assessment, the overall scores in both groups did not differ significantly. The courses themselves were positively evaluated by all participants. Herein, we present the first feasibility study of a web-based head and neck-ultrasound course for medical students. The methodology provides the potential for future changes in telemedical education and sustainable improvements in telemedical teaching and global intra-clinical and interdisciplinary patient care.

## 1. Introduction

The COVID-19 pandemic led to numerous changes and a rethinking of existing concepts in all areas of public life. Considering the hygiene and safety measures, social restrictions, and limited access to universities led to a widespread suspension of in-classroom teaching. At medical schools, numerous courses such as internships, hands-on training sessions, and medical clerkships could not be continued during the pandemic situation. New online-based concepts using video conferencing systems [1] and virtual classrooms [2] had to be established to keep up with existing courses and build new content from the ground up.

In particular, teaching manual clinical skills is a challenge in this context. However, ultrasound (US) courses are an integral part of clinical training for students and residents. Creating a curriculum and delivering US expertise to students requires significant time and human resources [3,4]. US is a sensitive, usually rapidly available, and radiation-free imaging technique for assessing soft tissues of the neck, such as cervical lymph nodes in inflammatory diseases and tumor follow-ups. Thus, US imaging plays an essential role in otolaryngology. In the hands of the skilled examiner, this form of imaging significantly accelerates clinical processes and contributes to enabling minimally invasive procedures for fast and efficient patient care [3,5]. Reliable handling of the technology is therefore mandatory in clinical practice. 

The examiner dependency [6] of sonographic findings highlights the need for practical experience and thus on-site training. Consequently, purely theoretical training does not seem to be sufficient in the context of education. The effectiveness of peer-assisted learning (PAL) approaches in US training has already been successfully demonstrated [7]. Additionally, in light of the constraints of in-person teaching, PAL-small group teaching was proposed considering the hygiene concepts, as switching to alternative concepts can be time-consuming and cost-intensive [8]. Radiology online courses for the interpretation of standardized acquired image data have been implemented successfully [9,10]. However, due to its dynamic nature, the performance of the US examination might place greater demands on the teacher and student in terms of direct communication and interaction. Therefore, our goal was to investigate whether a hands-on course can also take the form of web-based distance learning and whether it is comparable in quality and effectiveness to standard face-to-face formats. Telemedical guidance of inexperienced ultrasonographers such as paramedical personnel or medical students could be successfully realized [11,12,13,14,15]. However, comparison of the studies appears difficult as methodology, technical setup, anatomic area, research question, and objectives significantly varied. Online hands-on classes using video platforms during the COVID-19 pandemic have also been proposed in sports US training [16], but there are only scarce data concerning the learning success of those attending these courses compared to in-person classes.

This study aimed to establish an online video-based hands-on head and neck ultrasound (HANUS) course and evaluate this teaching method’s quality, effectiveness, and feasibility compared to in-person teaching. 

A further goal was to establish an interactive platform, taking into account the valid hygiene concepts, to be able to adapt swiftly to the needs of students and teachers for practice-oriented distance learning in the future.

In the following text, we describe the establishment of a video-based online ultrasound course for students with an explanation of the technical setup and didactic aspects as well as the evaluation of the course in comparison to the in-person format by self-assessment criteria and a score for an objective evaluation of learning outcome specifically designed for this purpose.

## 2. Materials and Methods

### 2.1. Study Design

**Figure 1:**
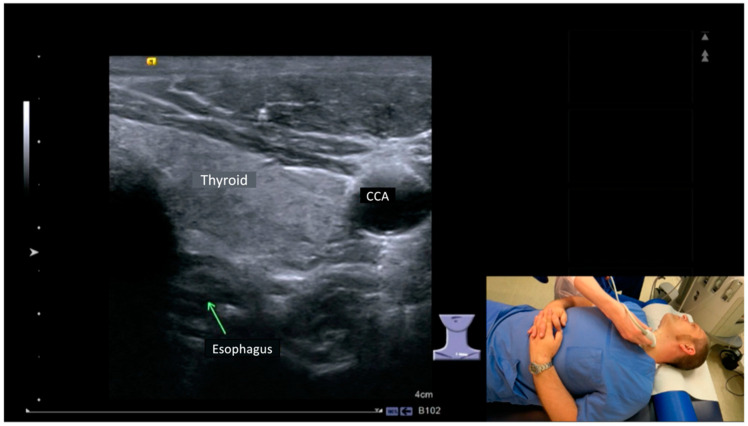
Excerpt from the step-by-step guide on how to perform a detailed HANUS examination. Ultrasound transducer and patient position were displayed in the lower right part of the screen, the standard planes were shown in the center of the screen, and the most important anatomical landmarks were labeled (ultrasound image showing the left lobe of the thyroid gland (thyroid), the esophagus, and common carotid artery (CCA)).

All students gave informed consent to participate in the study. Twenty-two students were randomly assigned to two groups, one group being taught in person and the other group in a video-based course. As part of our university college curriculum, the ultrasound course is designed for students within 45-min sessions. Therefore, each group was trained in pairs for 90 min. The students performed the HANUS examination on each other. A manual with a step-by-step guide on how to perform a detailed HANUS examination had been given to each student 48 h prior to the course start. This guide included the most important anatomical landmarks in HANUS, displaying the structure in the ultrasound image and the corresponding position of the patient and the transducer (Figure 1).

The course’s curriculum comprised a systematic technical introduction to the US system, basic steps for optimizing image quality and handling of the transducer, and a 4-step instruction for a detailed HANUS examination. The instructions were given by an experienced resident or consultant with at least five years of US experience in Oto-Rhino-Laryngology (ORL). 

The effectiveness and quality of our course were evaluated using a pretest–posttest design with a 4-point Likert scale self-assessment questionnaire (Table 1). Students were required to answer questions concerning their ability to locate anatomical landmarks before the course started. A questionnaire after completion of the course was used to evaluate whether learning objectives were met individually. Regarding the self-assessment score, a maximum of 36 points was possible. The questionnaire at the end of the course also addressed the students’ evaluation of the course formats. It considered aspects of didactic quality, such as time frame, clarity of teaching materials, tutor guidance, etc. This part of the questionnaire provided an opportunity to gain insight into students’ needs and expectations of the US course, compare course formats in this regard, and explore improvements for future courses.

In addition to the self-assessment questionnaires, we used another tool to measure the effectiveness of our course. After the training session, all participants were evaluated by a second examiner, a senior physician with a DEGUM (Deutsche Gesellschaft für Ultraschall in der Medizin) Level II degree.

For this purpose, an assessment score (Table 2) was used with a maximum achievable score of 50 points. Manual skills such as situs orientation, transducer handling, and knowledge of standard settings as well as optimization of the image by adjusting parameters such as focus, gain, frequency, etc., were assessed. Further criteria for the evaluation were the interaction with the patient, adjustment of the patient position, and systematic approach, as well as demonstration, measurement, description of the depicted anatomy, and potential abnormalities and pathologies of the structures.

Finally, statistical evaluation was performed using GraphPad Prism 7 (GraphPad Software, San Diego, CA, USA). We compared the self-assessment before and after the course in both groups using a two-way ANOVA. Correction for multiple testing was done using the Sidak method. The comparison of the groups with regard to the objective final learning success was made with the help of the *t*-test.

### 2.2. Technical Setup

#### 2.2.1. In-Person Teaching

For the group taught through in-person classes, a mobile US system (VINNO 6, VINNO, Suzhou, China) was set up in a room outside our outpatient clinic. The room size complied with the specifications regarding applicable distance regulations in the pandemic situation. The participants provided proof of vaccination or a negative COVID-19 rapid antigen test. An examination table was placed on the right side of the examiner. The US image was additionally displayed on a large screen allowing a better overview and compliance with the distance rules for teachers and students. 

#### 2.2.2. Video-Based Teaching

One US system (Siemens Acuson S2000, Siemens Healthcare GmbH, Erlangen, Germany) was set up in a room in our outpatient clinic, and another mobile system (VINNO 6, VINNO, Suzhou, China) was placed in a conference room outside the outpatient clinic. An examination table was positioned on the right side of the examiner. Each system was equipped with a 4K-capable video camera (GoProHero9, GoPro, San Mateo, CA, USA). The camera was positioned so that the examiner, the head and neck area of the patient, and the position of the receiving transducer were clearly visible.

**Figure 2:**
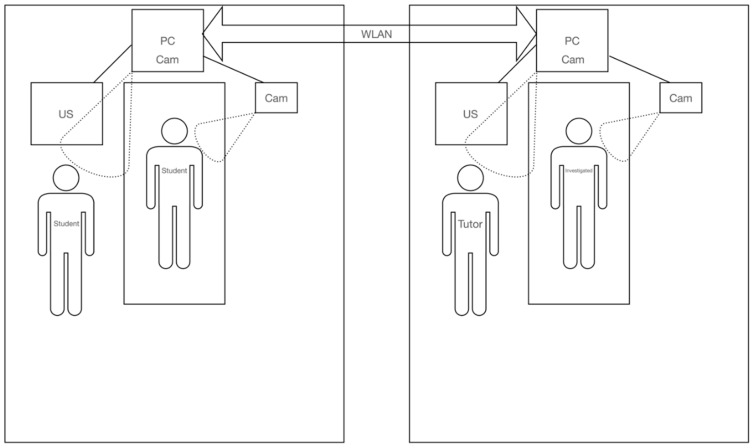
Technical setup for the video-based course. Screen display, patient position, and transducer position were transmitted to the corresponding system, and an audio connection was established via headphones and a condenser room microphone.

The US-video signal was transferred to a MacBook Pro (MacBook Pro 13”, 2020, Apple, Cupertino, CA, USA) or a Mac mini (Mac mini 2020, Apple, Cupertino, CA, USA) using the HDMI output of the US system and capture cards (Video capture, TKHIN, China). The other camera signals were fed in via USB-C/Thunderbolt. A virtual camera signal was created using OBS (Open Broadcaster Software, Open source software www.obsproject.com; accessed on 1 March 2021) to bundle all video signals and display them within a split-screen/cluster view on a large monitor in the sonography room of our outpatient clinic as well as the students´ room. This signal was used as the primary camera in Microsoft Teams (Microsoft, Redmond, Washington, DC, USA). An audio connection for the students was established using a condenser microphone (NTUSB, Rode Microphones, Sydney, Australia) and an external speaker (Bose Soundtouch 10, Bose, Framingham, MA, USA) connected to the computer. The instructor used a headset (Air pods, Apple, Cupertino, CA, USA) to communicate with the participants (Figure 2). For the students as well as for the instructor, the virtual camera signal included a split screen, which made the transducer position, patient position, ultrasound image, and the control panel of the ultrasound system comprehensible online for all participants. Aspects of handling, such as applied pressure of the transducer on the patient’s skin or coupling of the transducer and correct movement of the transducer along with the anatomical structures, could be evaluated indirectly based on image quality. The video-based course used the same curriculum as the in-person class. 

In each case, the students took turns assuming the role of the examiner or the patient and were accompanied and guided through the US exercises by the instructor.

## 3. Results

Characteristics of the student population are shown in Table 3. 

**Figure 3:**
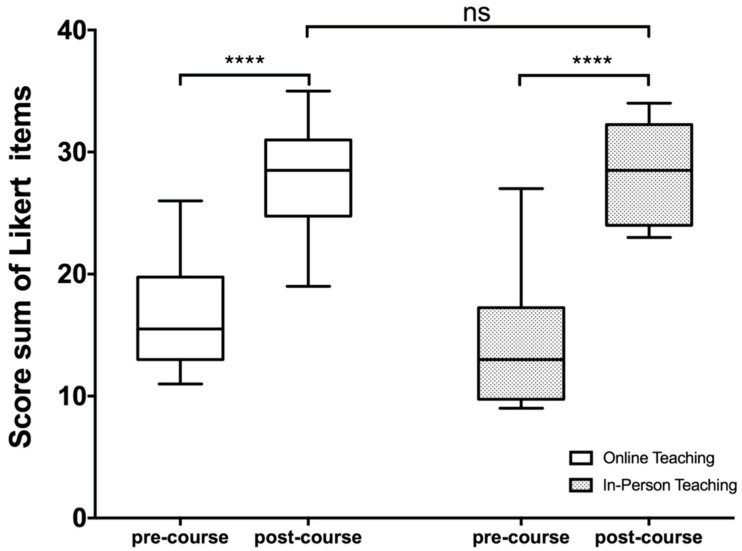
Participants’ self-evaluation of their ultrasound skills before (pre-course) and after (post-course) training. A 4-point Likert scale was used for self-assessment. The sum of the score values pre- and post-course for each group is shown in box plots. Two-way ANOVA after Sidak´s correction for multiple comparisons showed a significantly higher score after training in both groups (**** *p* < 0.0001).

Comparing pre- and post-training data, the whole population showed significant learning progress (Figure 3). Considering the self-assessment scores before the course, the participants expressed uncertainty in finding and naming the anatomic structures in the US examination. Following the training session, most participants of both groups could confidently find and display relevant anatomic structures of the head and neck area (Figure 3). 

**Figure 4:**
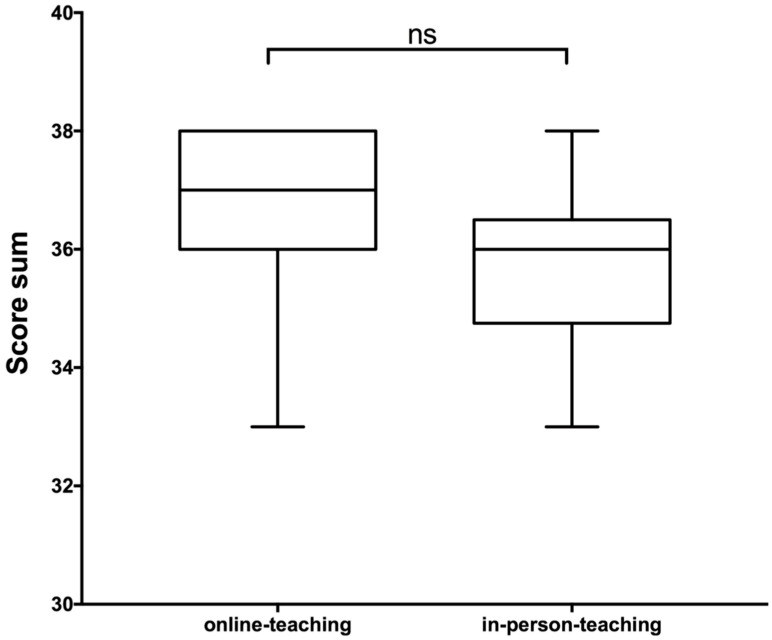
Score values reached in the final evaluation by an experienced US examiner according to the scoring system shown in Table 2. The *t*-test did not show any significant differences in the score values achieved in the objective assessment.

In the web-based course, participants improved from a median score value of 15.5 pre-course to a median score value of 28.5 after the training session. Regarding self-assessment, the group taught in face-to-face form showed an increase in the median score from 13 before to 28.5 after the training session. A significant difference in self-assessment after course participation between the two groups could not be found. Regarding the evaluation by the senior physician, we observed high score values in both groups. There was a slightly higher median score value reached by the video-based course (37) compared to the in-person class (36), but the overall scores in both groups did not differ significantly (Figure 4).

The course itself was positively evaluated by all participants of both courses with regard to personal learning success, time frame, course structure, and didactic aspects. A significant difference between the two groups concerning the aspects mentioned above could not be observed. 

## 4. Discussion

The present study investigated the quality and effectiveness of a HANUS course, comparing a video-based US course format with the so far “gold standard”, the in-person format.

Sonography represents a rapidly available, cost-effective, sensitive, and radiation-free imaging technique. As safe handling of this technology must be ensured in everyday clinical practice, US courses nowadays form a fundamental part of student training.

However, contact restrictions and concomitant limitations of in-person teaching during the COVID-19 pandemic presented a challenge to previously valid and common approaches focused on teaching practical skills.

Early studies on video-based US guidance originate from attempts to establish telemedicine US applications [11,12,13,14,15,16]. Efforts to create a competency-based assessment of surgical HANUS examinations, including web-based applications, have been cursorily described [17,18]. In these studies, US performances were recorded and edited in order to be evaluated by the examiner. The system did not support direct interaction or communication between the US user and the assessor. Ernst et al. pointed out the advantages of a web-based approach in structured reporting of head and neck US findings in medical education [19].

Nevertheless, there is no objective structured clinical examination (OSCE) as a standardized practice-oriented examination for HANUS to date. The effectiveness of ultrasound training in student education has already been demonstrated in many ways [20]. In addition to a better anatomical understanding, the development of practical clinical skills is also promoted by increased motivation [21]. Particularly in the context of the COVID-19 pandemic and teaching constraints, innovative approaches were needed to address student needs in ultrasound education. Our model and results need to be regarded in relation to these adaptations. One study demonstrated that ultrasound-inexperienced medical students can obtain basic practical skills in image acquisition after reviewing online modules that teach ultrasound techniques [22]. Meuwly et al. also used an online course and a virtual simulator. The computer mouse was used to operate the virtual transducer. The precision achieved during the simulation exercises was measured, and questionnaires assessing psychomotor skills were evaluated. The authors reported significant progress in the psychomotor domain of ultrasound diagnosis by integrating the online simulator for hands-on ultrasound learning in distance learning situations [23]. A prototype of a wearable augmented reality application to get familiar with the handling of ultrasound devices was also described [24]. Nevertheless, compared to our study, the approach remained asynchronous and without verification of the learned skills in an actual clinical setting.

The finding that instruction in ultrasound imaging does not depend on direct in-person contact between teacher and student but is also possible through live interaction via video conferencing tool was shown by DePhilip et al. [25]. However, in this study, only one-way live transmission of the ultrasound image from the instructor to the students was provided. The students, on the other hand, were not able to follow all steps of image acquisition. Direct interaction with synchronous assistance from the instructor was not provided. Zavitz et al. created a virtual point-of-care ultrasound (POCUS) course [26]. Video conferencing software was used for online lectures and demonstration of scanning techniques with the transmission of an ultrasound signal and demonstration of corresponding transducer handling. To familiarize the students with the physical/manual handling of the probe, students used the camera of their smartphones to aim at targets on the wall and thus simulate transducer movements. Olivares-Perez et al. used short online video courses and interactive virtual sessions to improve their understanding of the relevant ultrasound anatomy [27]. A comparison before and after instruction showed an increase in knowledge and confidence in the use of ultrasound. However, in this case, practical application experiences were also not possible for the students.

Herein lies another significant advantage of our study. The above setup with the double-sided display of patient position, transducer orientation, control panel settings, and ultrasound image allows direct interaction between instructor and student. This way, it is always possible to directly address problems or errors and thus ensure faster learning progress. Another strength of our work is the individually long hands-on time due to the small group size since, in many other in-person classes with less favorable instructor-to-student ratios, only one student ever has examination/active examination time at the ultrasound unit, while the rest of the group is less involved [28].

To our knowledge, this is the first web-based HANUS course described in the literature.

The structured clinical US examination provided in the script given to the students was evaluated as useful by all participants of both courses. The course structure, time frame, and teaching concept were also rated positively across the board. Consequently, our protocol for the structured examination shown here can serve as the basis for developing an OSCE for HANUS. This will result in better comparability of future studies and set new quality standards in head and neck ultrasonography.

Considering the individual learning success through video-based instruction, the methodology demonstrated in our study suggests that certified US courses (e.g., DEGUM) can also be web-based. Large distances from the training location with possibly long travel times for the course participants, a limited number of available US devices and instructors, and short intervals of effective hands-on time on the US device for the individual student are just a few reasons for insufficient access and limited learning success of currently available courses. With the help of our method, these obstacles can be overcome. However, positive effects are not limited to the use of technology in online US courses. The scalability of the methodology holds the possibility of offering practice-oriented courses across topics and disciplines to bridge even long distances and reduce travel time and costs. With the help of a decentralized, standardized teaching offer, the needs of the course participants can be addressed more individually, and the effective practical training time can be increased. The advantages and the potential of the methodology can be found beyond the teaching situation. The communication and monitoring interface established in our study represents a cost-effective and readily available tool for creating and expanding new intraclinical and global communication structures. Limiting factors for the widespread use of this technology must be discussed. Basic technical equipment and a stable Internet connection are crucial factors for reliable use. Especially in rural regions with still insufficient access to the Internet or inadequate digital infrastructure, this teaching model may still reach its limits. The financial aspect and the need for available ultrasound equipment and camera systems must also be taken into account. However, in our experience, the intelligent bundling of resources can result in more efficient teaching and even contribute to a significant improvement in the available infrastructure.

Several limitations of our study have to be considered. The subjects were “healthy” individuals without sonographic pathologies in the head and neck region, and the detection of pathologies was not part of the training. Therefore, we cannot draw any conclusions about the subjects’ abilities to transfer the acquired knowledge to clinical practice and pathological changes. This needs to be verified in further studies. Especially in the video-based instructional format, guidance in case of handling problems may seem challenging. However, the setup described in our manuscript and the possibility of online monitoring made it possible to address these aspects individually. While in the in-person format, handling issues can be addressed through direct manual demonstration, the video-based format poses a challenge to the instructor’s communication skills for descriptive instruction. However, in our experience, this was possible during instruction in both course formats.

Due to the hygiene measures, social restrictions, and the fact that our feasibility study has to be considered a pilot study, the number of participants (*n* = 22) is low, and the observation period was short. Nevertheless, studies concerning the effectiveness of one-day HANUS training sessions could show a significant increase in learning progress and level of comfort using the US technology [29].

The groups in our study were taught by the same resident instructor and were evaluated by the same senior physician. An estimation regarding the inter- or intra-observer bias is therefore not possible. However, regardless of group affiliation, we could not only find a high degree of learning success but also a highly positive evaluation of the didactic concept and the curriculum itself. This underlines the time efficiency of our concept.

Nevertheless, further studies are planned to evaluate the long-term effects of the training as well.

## 5. Conclusions

We herein present the, to our knowledge, first feasibility study and evaluation of a web-based head and neck ultrasound course. The present work forms the basis of future approaches to teach and develop technical skills of sonography in a web-based manner. No significant difference in individual learning success was shown in the comparison between classroom-based instruction and web-based remote instruction of students. With the technical equipment described here, online courses for teaching US skills are feasible in the future. Our preliminary work thus also provides the basis for future telemedical or telediagnostic applications. The methodology demonstrated holds potential for a sustainable improvement of teaching, clinical patient care, and global intraclinical and interdisciplinary networking.

## Figures and Tables

**Table 1:** Self-assessment questionnaire.

Self-Assessment Questionnaire					
Age					
Sex					
Clinical semester					
pre-course ultrasound experience	Yes	No			
If YES, pre-course knowledge gained through	Courses	Literature	Internships		
Self-assessment	Locating and visualizing the organ	1 = is not possible	2 = is rather possible	3 = Is possible	4 = is confidently possible
	Floor of mouth				
	Larynx				
	Trachea				
	Thyroid gland				
	Esophagus				
	Salivary glands				
	Vessels				
	Lymph nodes				
	Pathologies				
The time allocated for the course was sufficient	0	1	2	3	4
The teaching materials provided were sufficient	0	1	2	3	4
The comments of the tutors were helpful	0	1	2	3	4
The content of the course was sufficient	0	1	2	3	4
The tasks were clearly formulated	0	1	2	3	4
The teaching materials provided were clearly structured	0	1	2	3	4
The learning content did not overwhelm me	0	1	2	3	4
The group size was comfortable	0	1	2	3	4
The explanations of the tutors were well understandable	0	1	2	3	4
My questions were answered sufficiently	0	1	2	3	4
The feedback on my skills was sufficient	0	1	2	3	4
I consider the course format suitable for knowledge transfer	0	1	2	3	4
It would be useful to extend this course to other organ systems	0	1	2	3	4

**Table 2:** External assessment score.

Assessment Category	Score Points	
Manual skills	max. 8 points	
Situs orientation	0–2	Landmarks and guiding structures are recognized: 0 = no, 1 = partially, 2 = relevant structures can be named
Transducer positioning	0–2	0 = no orientation, 1 = partial orientation, 2 = complete orientation
Adjusting the focus position	0–2	0 = no focus, 1 = partial focus, 2 = focus is adjusted
Adjusting gain	0–2	0 = no gain, 1 = partial gain, 2 = gain is adjusted
Interaction with the patient	max. 4 points	Score: 0 = There was no interaction between investigator and patient 1 = The investigator has informed the patient about the planned examination 1 = The investigator has encouraged the patient to cooperate 1 = The investigator explained the results to the patient 1 = The investigator has taken an introductory medical history
Patient positioning	0–2	0 = no adjustment of patient position, 1 = partial adjustment of patient position, 2 = patient position is adjusted and properly optimized
Systematics of the examination	0–2	0 = no systematics, 1 = partial systematics, 2 = good systematics
Visualization of the organs	max. 20 points	
Measuring	0–4	0–4 Organs or structures are measured correctly
Demonstration	0–16	0–16 Organs are displayed
Explanation of the anatomy shown	max. 18 points	
Normal findings	0–14	0–16 Organs are examined
Pathologies	0–4	0–4 Pathologies are assessed; if no pathology, then 4 points
Score total points	max. 50 points	

**Table 3:** Characteristics of the study population (*n* = 22). Median, maximum, and minimum (brackets) or total number of participants.

	Online Course	In-Person Course
Age	23 (21–29)	22.5 (22–30)
Gender (female/male)	6:6	7:3
Semester	8 (6–10)	6 (6–8)
Ultrasound-knowledge/experience pre-course (courses, internships, literature)	66.6%	40%

## Data Availability

The data presented in this study are available on request from the corresponding author.
